# Complete laparoscopic cholecystectomy for a duplicated gallbladder: A case report

**DOI:** 10.1097/MD.0000000000018363

**Published:** 2020-01-03

**Authors:** Dong-Kai Zhou, Yu Huang, Yang Kong, Zhou Ye, Li-Xiong Ying, Wei-Lin Wang

**Affiliations:** a Department of Hepatobiliary and Pancreatic Surgery, Department of Surgery, The Second Affiliated Hospital, School of Medicine, Zhejiang University; b Key Laboratory of Precision Diagnosis and Treatment for Hepatobiliary and Pancreatic Tumor of Zhejiang Province; c Clinical Research Center of Hepatobiliary and Pancreatic Diseases of Zhejiang Province; d Division of Hepatobiliary and Pancreatic Surgery, Department of Surgery; e Department of Pathology, The First Affiliated Hospital, School of Medicine, Zhejiang University, Hangzhou, Zhejiang Province, China.

**Keywords:** case report, cholangiography, duplicated gallbladders, histopathology, laparoscopic cholecystectomy

## Abstract

**Introduction:**

Duplication of the gallbladder (GB) is a rare congenital abnormality occurring in 1 in 4000 to 5000 births. Three types have been reported: type I (split primordial GB), type II (2 separate GBs with their own cystic ducts), and type III (triple GBs drained by 1 to 3 separate cystic ducts). Patients with a duplicated GB are usually asymptomatic and are sometimes not diagnosed on preoperative imaging, which might increase the difficulty and risk of cholecystectomy. The key to successful treatment is total removal of the duplicated GB to avoid the recurrence of disease. Intraoperative cholangiography is recommended for identifying and resecting duplicated GBs. The final diagnosis depends on the histopathology.

**Patient concerns:**

A 62-year-old woman had recurrent upper abdominal pain and nausea for 1 year, with no fever, jaundice, or other symptoms. An ultrasound of the abdomen indicated polyps in the GB. Computed tomography (CT) revealed moderate dense structures attached to the wall of the GB and an unusual 47 × 21 mm elliptical structure with an extra tubule located above the main GB.

**Diagnosis:**

A diagnosis of duplicated GB was made based on the histopathology.

**Interventions:**

The patient underwent a laparoscopic cholecystectomy with total removal of the duplicated GB.

**Outcomes:**

The patient's postoperative course was uneventful and she was discharged from the hospital on the second postoperative day. She had no upper abdominal pain at the 6-month follow-up.

**Conclusion:**

Duplicated gallbladder is a rare congenital biliary anatomy, which is usually asymptomatic and sometimes cannot be diagnosed on preoperative imaging. With gallbladder disease, the duplicated GBs should be removed totally; a laparoscopic approach should be attempted first and cholangiography is recommended to aid in identifying and resecting the duplicated GBs. The final diagnosis depends on the histopathology. There is still insufficient evidence on the need to remove duplicated GBs found incidentally.

## 1 Introduction

Abnormal biliary anatomy is frequently observed during surgery. A duplicated gallbladder (GB) is a rare congenital abnormality occurring in 1 in 4000 to 5000 births.^[1]^ Duplicated GBs can develop gallstone disease and biliary colic, but seldom develop into carcinoma.^[2]^ Duplicated GBs can be classified into 3 types: type I, split primordial gallbladder; type II, 2 separate gallbladders with their own cystic ducts; and type III, triple gallbladders draining by 1 to 3 separate cystic ducts. Type II duplicated GB is the most common, with each GB draining into the common bile duct through a separate cystic duct.^[3]^ A duplicated GB is seldom detected preoperatively, which increases the difficulties and risks in biliary surgery, including the possibility of conversion to open surgery, biliary injury, and other postoperative complications. Here, we report a challenging laparoscopic cholecystectomy for a duplicated GB in a woman who presented with biliary colic and polyp disease.

## 2 Case report

**Figure 1:**
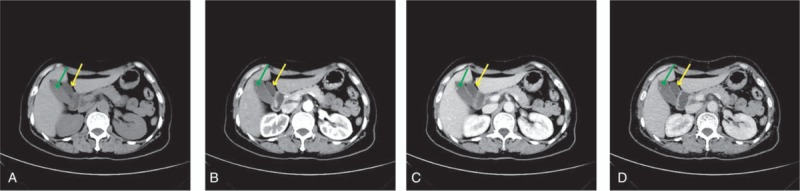
CT revealed an unusual elliptical dense mass (green arrow) with an extra tubular structure located above and medial to the native GB (yellow arrow), which measured about 47 × 21 mm^2^. CT = computed tomography, GB = gallbladder.

**Figure 2:**
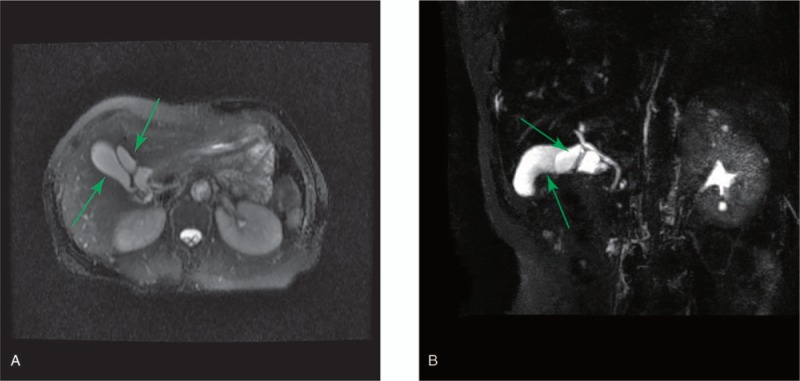
MRCP indicated 2 separate GBs (green arrow) with their own cystic ducts connecting to the biliary tree. One contained bile of varying density and had a nonhomogeneous density structure. GB = gallbladder, MRCP = magnetic resonance cholangiopancreatography.

A 62-year-old woman had recurrent upper abdominal pain and nausea for 1 year, with no fever, jaundice, or other symptoms. Ultrasound of the abdomen suggested polyps in the GB. Gastroscopy suggested chronic superficial gastritis. Abdominal contrast-enhanced computer tomography (CT) revealed moderate dense structures attached to the GB wall and an unusual 47 × 21 mm^2^ elliptical structure with an extra tubule located above the main GB (Fig. 1A–D). Magnetic resonance cholangiopancreatography (MRCP) indicated 2 separate GBs with their own cystic ducts to the biliary tree; one of these contained bile of varying density (Fig. 2).

**Figure 3:**
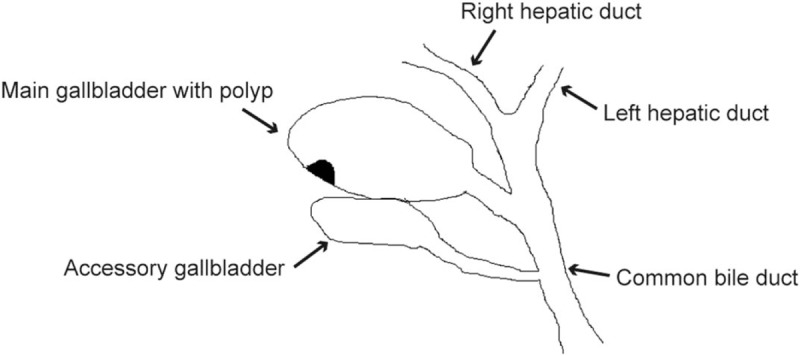
Illustration of our case: the double gallbladders with polyp.

**Figure 4:**
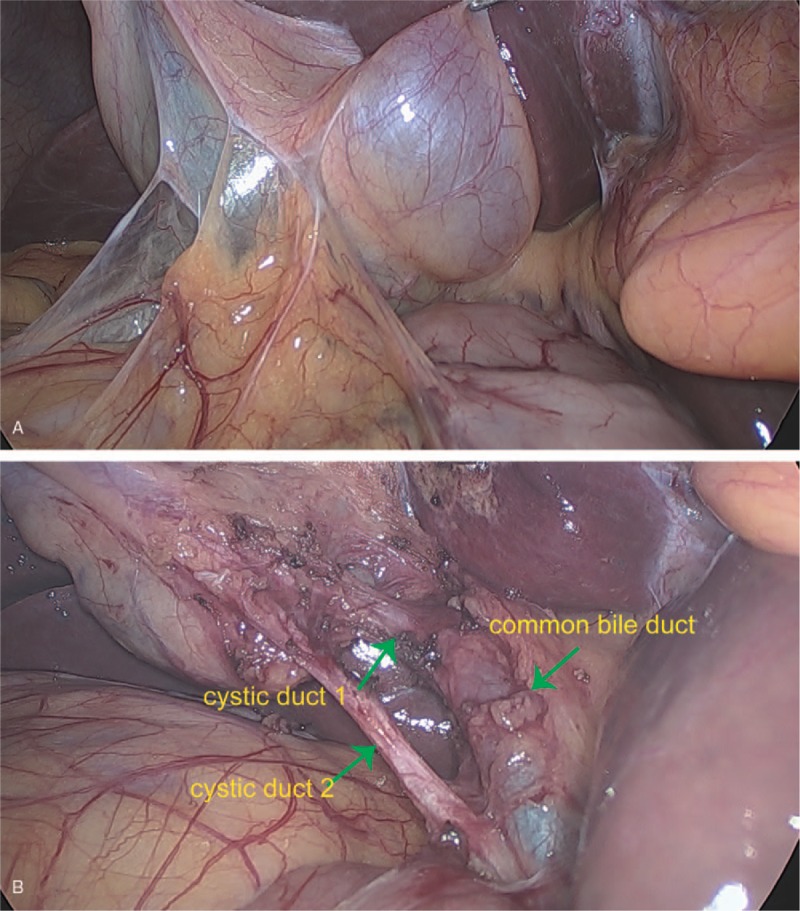
The surrounding structures were dissected carefully and the cystic duct of each GB was exposed (green arrow). GB = gallbladder.

**Figure 5:**
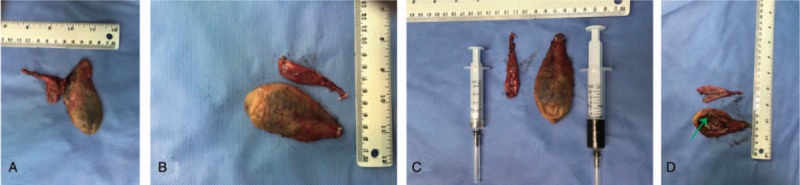
Two GBs were successfully resected. (A) We separated the 2 GBs and extracted bile from both. The bile was dark green in one and white in the other (B and C). The polypoid lesion was identified in the larger of the resected GBs (green arrow) (D). GB = gallbladder.

**Figure 6:**
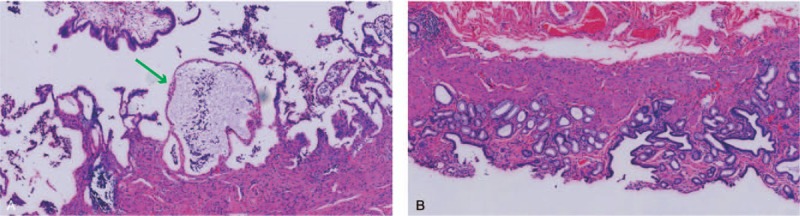
Histopathology indicated that one GB had a polyp (green arrow) and features of chronic cholecystitis; both lacked evidence of neoplasia. GB = gallbladder.

The patient underwent a laparoscopic cholecystectomy. At surgery, twin GBs were found in a common GB fossa adhering to the surrounding structures; it was H-type according to the Harlaftis classification (Fig. 3). The duodenum, stomach, and transverse colon were carefully examined and separated. The surrounding structures were dissected carefully and each cystic duct was exposed (Fig. 4). Both cystic ducts and their blood vessels were divided and clipped, and the 2 GBs were successfully resected (Fig. 5A). We opened both GBs and removed dark green bile from one and white bile from the other (Fig. 5B and C). The polypoid lesion was identified as the largest resected GB (Fig. 5D). The final histopathology revealed 2 separate GBs, one with polyps and features of chronic cholecystitis; there was no evidence of neoplasia in either (Fig. 6). No postoperative complications occurred and the patient was discharged on the second postoperative day. The patient remained free of upper abdominal pain at the 6-month follow-up.

## 3 Discussion

Duplication of the GB is a rare congenital aberrance occurring in 1 in 4000 to 5000 births.^[1]^ It was first reported in 1674 during an autopsy. In 1911, Sherren documented the first case of a duplicated GB in a living person.^[4,5]^ Several authors have documented different anatomical variations of duplicated GBs, mainly in case reports.^[6,7]^

**Figure 7:**
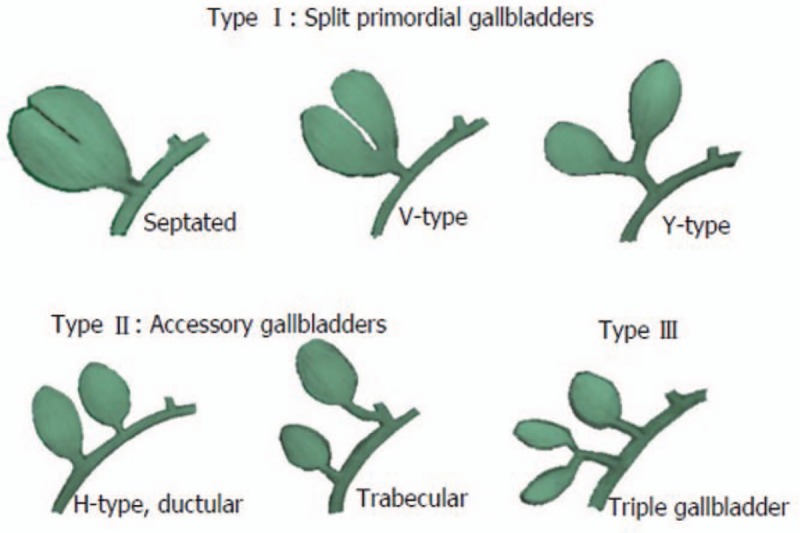
The Harlaftis classification of anatomical variation of accessory gallbladders.^[2]^

The Harlaftis classification is still used most widely and classifies the duplicated GBs anomalies into 3 types. In type I, the primary GB is split and includes partially split (septated), partly split (V-shaped with 2 GBs joined at the neck), and completely split (Y-shaped with 2 cystic ducts joining into a common cystic duct). All of these subtypes drain into the common bile duct through a single cystic duct. These gallbladders are usually next to each other and share a GB fossa. In type II anomalies, the most common form, 2 separate GBs drain into a common bile duct through independent cystic ducts (H-type), or one of the cystic ducts drains into the right or left hepatic duct (trabecular type) (Fig. 7).^[2,3]^ Kawanishi et al^[8]^ reviewed 148 cases of duplicated GB and concluded that the H-type was the most common, accounting for nearly half of the reports. A type III anomaly is one that does not fit either type I or II, involving triple GBs draining through 1 to 3 separate cystic ducts.^[5,8,9]^ Cases of duplicated intrahepatic GBs have also been reported. Won et al^[10]^ reported a duplicated gallbladder in an intrahepatic location mimicking a cystic intraductal papillary neoplasm of the bile duct. Roeder et al^[11]^ reported a triplicated GBs, with 2 removed surgically, and the third indicated by preoperative T-tube cholangiogram, but not identified at surgery; it was assumed to be intrahepatic. Schroeder and Draper^[12]^ reported a triplicated GBs, 2 of which were identified preoperatively; the third was found to be intrahepatic during the operation.

Although the rate of diagnosis of duplicated GBs is increasing with improvements in imaging methods, there have been case reports of duplicated GBs missed on routine preoperative imaging.^[13–15]^ Ultrasound and CT usually cannot provide sufficient visualization of the biliary tract to detect duplicated GBs reliably, while MRCP is appropriate when duplicated GBs are suspected.^[15–17]^ It is very important for surgeons to take thorough measures to detect duplicated GBs and to perform a careful biliary investigation intraoperatively when duplicated GBs are suspected.

Once a duplicated GB is identified, most surgeons agree that the GBs should be removed totally to avoid postoperative complications in patients with gallbladder disease.^[4,14,15,18]^ There have been reports of patients returning with biliary symptoms after incomplete resection of a duplicated GB.^[13,19,20]^ Reinisch et al^[21]^ reported a patient who underwent an extra laparoscopic cholecystectomy because of acute cholecystitis of the remaining duplicated GB, which was not detected originally. Juillerat et al^[22]^ reported a child with duplicated GBs who underwent surgery for symptomatic cholelithiasis and was found to have heterotopic gastrointestinal mucosa and pancreatic microclusters. Other postoperative complications due to a remaining duplicated GB include cholecystitis, empyema, cholecystocolic fistula, torsion, papilloma, and carcinoma.^[4,11,14,23–27]^ Cholangiography during cholecystectomy reduces the possibility of extra bile duct injury by 30%.^[15,20,28]^ Hence, intraoperative cholangiography is recommended in cases of duplicated GBs to assure complete resection of the duplicated GBs and avoid injury to the biliary trees.^[28–30]^ Although the outcomes and complications vary, surgeons agree that duplicated GBs should be removed totally; a laparoscopic approach should be the initial choice and cholangiography is recommended to aid in identifying and resecting the duplicated GBs. The final diagnosis depends on the histopathology. Note that there is still insufficient evidence regarding the need to remove duplicated GBs found incidentally; we recommend treating only duplicated GBs in patients with gallbladder disease.

## 4 Ethical statement

Written informed consent was obtained from the patient. Ethical approval was obtained from the Ethics Committee of the First Affiliated Hospital, School of Medicine, Zhejiang University, China, in accordance with the ethical guidelines of the 1975 Declaration of Helsinki.

## Author contributions

**Conceptualization:** Dong-Kai Zhou, Yu Huang.

**Resources:** Li-Xiong Ying.

**Supervision:** Weilin Wang.

**Writing – original draft:** Dong-Kai Zhou, Yu Huang.

**Writing – review & editing:** Yang Kong, Zhou Ye, Weilin Wang.
